# “Grace Under Pressure”: How CEOs Use Serious Leisure to Cope With the Demands of Their Job

**DOI:** 10.3389/fpsyg.2020.01453

**Published:** 2020-07-03

**Authors:** Emilia Bunea

**Affiliations:** School of Business and Economics, Vrije Universiteit Amsterdam, Amsterdam, Netherlands

**Keywords:** CEOs, serious leisure, leader resources, leader stress, executive job demands

## Abstract

How chief executive officers (CEOs) use their leisure to help respond to the demands of their job is important for themselves, their employees, and their organizations. This study shines light on this hardly explored subject by focusing on CEOs of major US companies and their “serious leisure,” the goal-oriented pursuit of a non-work passion. Serious leisure is increasingly practiced by the population at large as well as by top leaders. This study is based on 16 interviews with “serious leisurite” CEOs of Fortune 500, S&P 500, or comparable organizations. Novel insights are brought into the ways in which CEOs believe their passionate non-work pursuit supports not only coping with the strain of the top job but also optimal functioning in it, as well as into how they perceive the demands of the CEO role. This work contributes to research on leader personal resources and leader effectiveness, executive job demands, as well as to the leisure-based recovery literature.

## Introduction

When Jeff Kindler, chief executive officer (CEO) of Pfizer, abruptly resigned in 2011, he blamed the “24/7 struggle” to meet the high and conflicting demands of his many stakeholders (Lemer, 2011, p. 1). In a 2018 interview, Elon Musk was on the brink of tears while describing the “excruciating personal toll” of leading Tesla (Gelles, 2018, p. 1). Although rare compared to the usual, carefully scripted self-portrayals of CEOs as energetic and in control (Gray and Densten, 2007; Pollach and Kerbler, 2011), confessions like these remind us that the leaders of the corporate world do sometimes become overwhelmed by the demands of their job.

Why should we care? As public opinion has it, CEOs get handsomely rewarded for any inconvenience their jobs may present. Moreover, several studies show that managers’ mental health is not worse than that of non-managers (Ganster, 2005; Skakon et al., 2011; Sherman et al., 2012; Li et al., 2018). One could debate how applicable these studies, based on entry-level to mid-level managers often working in government or the military, may be to the corporate executive suite, but such debates would take our focus away from the fact that, when it does occur, CEO stress has far-reaching consequences for the company’s results (Hambrick et al., 2005a; Siren et al., 2018) and for the quality of their leadership (Hambrick et al., 2005b; Sprague et al., 2011; Wirtz et al., 2017). Given that job-related stress arises from an imbalance between the demands of the job and the resources available to do it (Bakker and Demerouti, 2017) and since executive job demands are “qualitatively different” from job demands at other levels of the organization (Hambrick et al., 2005a, p. 474), it is thus important to understand how CEOs experience the challenges of their job and how they cope with the stress involved. Moreover, examining leaders’ mental health should go beyond stress to considering “positive attributes such as optimism, hope, vigor, and self-efficacy; the presence of such attributes may be more important than the absence of mental health challenges” (Barling and Cloutier, 2017, p. 399). Yet the question of leaders’ mental health has hardly been addressed in empirical studies, be it because we see CEOs as “hardened individuals capable of handling substantial stress” (Siren et al., 2018, p. 954) or because it is notoriously hard for researchers to access CEOs’ inner life (Mueller and Lovell, 2015).

This paper explores how CEOs who have a passionate non-work interest (a “serious leisure” interest) perceive the role of their serious leisure in helping them cope with the demands of their job. The choice of focusing on “serious leisurites” was not only because talking about a personal activity that is dear to their heart was more likely to make CEOs drop their “corporate speak” defenses but also because there has been little research attention to how serious leisure interacts with work in general, let alone executives’ work. With society-wide serious leisure participation continuing to grow, from amateur music (Bonde et al., 2018) to “career volunteering” (Cantillon and Baker, 2019) to marathon running (Vitti et al., 2019), and with serious leisurite CEOs no longer a rare occurrence, a qualitative exploration of this novel connection, how top leaders see the role of their serious leisure in responding to their job demands, is warranted.

To answer the research question, interviews were held with 25 CEOs of S&P 500 or comparable organizations (median headcount: 16,000). As the interviews revealed that only 16 among the interviewees (median headcount: 19,000) have a serious leisure interest, these 16 interviews constituted the final sample. Although the intensity of executive job demands is subjective (reflecting the incumbent’s perception of how difficult the top job is) and depends on many factors in addition to company size, large public companies can be expected to place higher demands on their CEOs than smaller or private organizations (Hambrick et al., 2005a). Given the size of their companies, the CEOs in our sample thus likely represent an extreme case of executive job demands, which is a good place to start when qualitatively exploring new theoretical territories (Eisenhardt, 1989).

The analysis of the data has brought up novel insights with regard to how CEOs perceive the demands of their job, including those pertaining to transformational leadership, how they see the stress involved, and how they believe their serious leisure interests uniquely help them both manage this stress and build substantial personal resources needed for optimal functioning in the CEO role. Thus, this study contributes to research on leader psychological resources (Barron et al., 2000; Chen, 2015; Rego et al., 2019), upper echelon theory, and specifically executive job demands (Hambrick et al., 2005a) and to studies of work recovery through leisure (Sonnentag et al., 2017).

## Theoretical Background

### CEO Job Demands and Personal Resources

The upper echelon theory proposes that top executives’ idiosyncratic characteristics, perceptions, and dispositions have a significant impact on organizational outcomes (Hambrick, 2007) and that an important moderator for this relationship is represented by executive job demands, defined as “the degree to which a given executive experiences his or her job as difficult or challenging” (Hambrick et al., 2005a, p. 474). More generally, the job demands–resources (JD–R) theory defines job demands as those aspects of the job that require sustained effort and thus exact psychological and/or physiological costs from the jobholder. High job demands are associated with high risk of strain, but the availability of job resources moderates this relationship, acting as a buffer (Demerouti et al., 2001). Recognizing that “stress” is a concept that has been variously defined and that can encompass a wider array of symptoms, this study follows our interviewees’ representation of it as emotional exhaustion and/or anxiety, and therefore it uses the terms emotional exhaustion, stress, and strain interchangeably.

Compared to job demands in the JD–R model, executive job demands represent a phenomenological, not an objectively measurable, construct and seem to already take into account the (perceived) job resources available since perceived job difficulty would likely represent a “net result” of job demands minus job resources. While the JD–R model represents the general background for this paper, the executive job demands concept is specifically adopted as closest to the interviewees’ sensemaking with regard to the challenges of their role.

Later refinements of the JD–R model added another important moderator of the job demands–burnout relationship: personal resources (Bakker and Demerouti, 2017). Moreover, according to the JD–R theory, high resources (job and personal ones) are also associated with high job motivation especially when job demands are also high: in other words, resources are most appreciated when they are highly needed (Bakker and Demerouti, 2017).

Examples of personal resources found to moderate the risk of emotional exhaustion and/or to promote motivation when job demands are high are optimism, self-efficacy, and self-esteem (Huang et al., 2016), psychological capital (a set of positive psychological resources consisting of hope, self-efficacy, resilience, and optimism) (Luthans and Youssef-Morgan, 2017; Madrid et al., 2018), mindfulness (Grover et al., 2017), “tenacity” (persisting toward one’s goals) (De Clercq and Belausteguigoitia, 2017), and “core self-evaluations” (a construct composed of self-esteem, locus of control, general self-efficacy, and emotional stability) (van Doorn and Hulsheger, 2015). What is missing from this wealth of quantitative findings is the voice of the jobholders themselves and therefore a more nuanced understanding of how they use personal resources to cope with the demands of their work, where coping represents “the thoughts and behaviors used to manage the internal and external demands of situations that are appraised as stressful” (Folkman and Moskowitz, 2004, p. 745). Moreover, the role of personal resources in relation to executive job demands has not been examined. While I believe, unlike some observers of the C-suite, that CEOs are also people and therefore the accumulated research on personal resources and job demands is, to some extent, also applicable to them, the subjective and the different nature of executive job demands (Hambrick et al., 2005a) justifies the need for a dedicated investigation of how CEOs perceive the demands of their role and how their personal resources can help them respond to such demands.

In their influential paper on executive job demands, Hambrick et al. (2005a) propose (while admitting that empirical insight is yet to come) that, unlike in the general JD–R model, top executives might partly create their own job demands by setting high aspirations for their own performance. An important dimension of these aspirations is arguably represented by the CEOs’ expectations of themselves as leaders.

### CEOs’ Expectations of Themselves as Leaders

Individuals hold cognitive schemas specifying what they expect leaders are like and how they behave, known as “implicit leadership theories” (ILTs). For the population at large, these conceptualizations have changed little over the past two decades, with “true” leaders still seen, for example, as highly dedicated to their role, strong, and charismatic (Offermann and Coats, 2018). One can expect CEOs’ ILTs to be at least partially influenced by the generally shared ones, but there is little empirical insight thereon. CEOs do often share their views of leadership in public interviews (e.g., Gordon and Martin, 2019). However, these outwardly expressed views are arguably as much influenced by prevailing discourses of what constitutes “good leadership” as by the CEOs’ own unfiltered sensemaking. In turn, discourses of “good leadership” emphasize behaviors that are most often associated with “positive” forms of leadership such as transformational, charismatic, authentic, or ethical leadership (Alvesson and Einola, 2019). While there has been vigorous scholarly questioning of the value added by authentic, ethical, and servant leadership concepts over and above transformational leadership (Hoch et al., 2018), most conceptualizations of “good leadership” include at least part of the four tenets of transformational leadership behavior (inspirational motivation, idealized influence, intellectual stimulation, and individual consideration), and this is reflected in leadership training programs and textbooks.

Expectations from leaders are also informed by the phenomenon known as the “romance of leadership,” the tendency to attribute an exaggerated, larger-than-life role of the leader in the success or in the failure of their company (Meindl and Ehrlich, 1987), yet most research efforts have focused on the followers’ romance of leadership perceptions, with little known about the leaders’ own romance of leadership notions (Bligh et al., 2011) and how these notions influence their expectations of themselves. A large-scale study of Australian executives (the majority working in organizations of 500 or fewer employees) found that they engaged in “self-deception” triggered by romance of leadership notions (Gray and Densten, 2007), which would suggest self-deception as a way of bridging the gap between the leaders’ perceived own leadership abilities and their own romantic notions of leadership.

One important dimension of ILTs (dedication), when joined to the romantic expectation of heroic deeds by the leader, may converge into the expectation that leaders self-sacrifice for the good of the collective. Research has shown that leaders who engage in self-sacrificial behavior are seen as more effective by their followers (De Cremer and van Knippenberg, 2004; Van Knippenberg and Van Knippenberg, 2005) and that leaders who are higher in power (arguably the case of CEOs) may be more inclined to self-sacrifice than lower-power leaders (Hoogervorst et al., 2012).

In this context, we know little about how CEOs’ expectations of themselves as leaders influence their perceived executive job demands.

### Leader Psychological Resources and Leader Effectiveness

Research regarding the role psychological resources play in leaders’ effectiveness has mostly focused on psychological capital, found to alleviate the strain caused by high job demands (Hur et al., 2016; Sheng et al., 2019) and, alongside other positive resources such as mindfulness, to predict the well-being of leaders (Roche et al., 2014). When leaders’ psychological capital is high, the quality of leader–member exchange increases, in turn promoting high psychological capital for followers (Chen et al., 2019). Global leaders’ psychological capital buffers the negative effects of geographical distance on the relationship with their followers (Story et al., 2013).

This paper mentions “leader” and “CEO” interchangeably, although it fully subscribes to the view that conceptualizations of leadership should be de-coupled from formal positions of management. The belief that these interviews with CEOs are also relevant for the broader leadership literature stems firstly from the fact that the interviewed CEOs themselves show a preoccupation for, and talk about, leadership and leadership development (theirs and others’), secondly from the high likelihood that, given their formal positions and work experience, these CEOs would, in fact, engage in leadership in their day-to-day work (DeRue and Ashford, 2010), and thirdly from the fact that CEOs of S&P 500 companies are highly visible and therefore have a word to say in the shaping of prevailing discourses on leadership, which in turn inform the construction of leadership by other (would be) leaders and their (potential) followers.

### Serious Leisure and Personal Resources

Grounded in the Conservation of Resources Theory (Hobfoll, 1989), an ample body of research documents how off-work recovery experiences restore personal resources depleted by work (Sonnentag et al., 2017). The initial list of four types of recovery experiences (psychological detachment from work, relaxation, mastery, and control) was subsequently extended with the experience of pleasure and enjoyment (Sonnentag and Fritz, 2007). These experiences promote the creation of personal resources such as self-efficacy, positive affect, and relevant skills or (in the case of detachment) protect against further resource loss (Sonnentag and Fritz, 2007).

While most leisure activities can provide some form of recovery experience, *serious* leisure is ideally positioned in terms of the range and the strength of recovery experiences and personal resources it promotes. Serious leisure represents “the systematic pursuit of an amateur, hobbyist or volunteer activity that is sufficiently substantial and interesting for a participant to find a ‘career’ there in the acquisition and expression of its special skills and knowledge” (Stebbins, 1982, p. 3). By definition, serious leisure is different from casual leisure based on six characteristics: first, it involves a significant effort in mastering a specific skill or knowledge; second, it requires perseverance as serious leisure participants occasionally face setbacks, fatigue, and other challenges; third, it has its unique ethos, a social world around the serious leisure pursuit, that has its own beliefs, values, and norms (Stebbins, 1982); fourth, it develops a strong serious leisure identity; fifth, it often leads to a leisure “career” that progresses during a person’s life through “special contingencies, turning points, and stages of achievement and involvement” (Stebbins, 2007, p. 11); and lastly, it generates several types of enduring benefits, the most frequent of which are (a) self-actualization and personal growth, (b) confidence and enhancement of self-image through the display of unique skills, capacities, and knowledge, (c) feelings of connection and belonging supported by the pursuit’s social world, (d) self-expression (expressing one’s abilities and individuality), (e) renewal, regeneration, and recovery, (f) mastery, competence, and feelings of accomplishment, and (g) happiness and subjective well-being (Major, 2001; Stalp, 2006; Bendle and Patterson, 2009; Kim et al., 2015).

This powerful cocktail of personal resources built by serious leisure suggests that it can play an important role in buffering the burnout risk associated with high job demands, yet we know little about how serious leisurites perceive this relationship. Importantly, beliefs about coping with stress through one’s leisure do have a stress-buffering effect (Iwasaki and Mannell, 2000). Therefore, a qualitative exploration of how serious leisurites believe that their passion helps them cope with the demands of their jobs can offer valuable insights.

## Materials and Methods

### Sample and Data Collection

As little is known about how CEOs perceive the role of their passionate non-work interests in responding to the demands of their jobs, I opted for a qualitative approach to this investigation. First, I purposefully built the sample as follows: as a former corporate CEO myself, I started by sending LinkedIn invitations to connect to all CEOs of the top 1,000 US companies listed on Glassdoor.com who had a LinkedIn profile, as well as to all CEOs of S&P 500 and/or Fortune 500 companies (if not already included among the 1,000 CEOs mentioned earlier) who were present on LinkedIn. The invitations used the standard template offered by LinkedIn, with no customized text. After expanding my network of CEO connections in this manner, I sent requests for interviews to the 147 CEOs among these connections who led companies with over 5,000 employees.^1^ The interview invitations mentioned the intention to discuss the CEOs’ passionate non-work interests (if any) and how they interacted with their leadership. A total of 29 CEOs responded positively (20% response rate), with 25 semi-structured interviews being eventually conducted.

During the interviews, I assessed whether the CEOs’ non-work interests qualified as serious leisure by broadly reviewing the six properties that differentiate serious from casual leisure (Gould et al., 2008). I found out that 16 of the interviewed CEOs had a non-work interest that could qualify as serious leisure, and therefore they represented the final interview sample. Their organizations count between 3,500 and 79,000 employees^2^ (median 19,000 employees) and their annual revenues being between $1 billion and $137 billion (median $4.3 billion). At the time of our interviews, three out of the 16 interviewees had recently stepped down from the CEO position (with the longest time from their last day in the office to our interview being 10 months). Of the 16 CEOs, 11 lead companies listed in the S&P 500, the Fortune 500, or the FTSE 100 indices, with the remaining five companies having a median headcount of 22,000. By comparison, the median headcount of companies listed in the S&P 500 index (the largest public US organizations) at the end of 2017 was 20,500^3^. The interviewees’ companies represent a wide variety of industries^4^.

As mentioned earlier, given the size of their companies, these CEOs likely represent an “extreme case” of serious leisurite executives and their job demands, which makes their narratives especially valuable because they can surface insights that would be harder to attain under more regular conditions (Eisenhardt, 1989). Thus, although objectively small, the sample of 16 interviews with CEOs who have a passionate non-work interest is suitable for the analytical, rather than statistical, generalization targeted by qualitative research (Yin, 2013).

Table 1 presents the list of interviewed CEOs (who were assigned fictional names for anonymity) and their serious leisure interests.

**TABLE 1:** Interviewed CEOs and their serious leisure interests.

CEO name (fictional)	Serious leisure interest	CEO name (fictional)	Serious leisure interest
Jason	Cycling	Steven	Ice hockey
Richard	Soccer	James	Music
Paul	Tennis	Mary	Martial arts
Larry	Football coaching	Robert	Handcrafting greeting cards
Thomas	Woodwork	Kenneth	Running
Jack	Golf	Anthony	Music, singer–songwriter
Kevin	Golf, airplane piloting	Susan	Running
Bill	Airplane piloting	Laura	Horse riding

The interview protocol cast a wider net, starting from the initial broad question: “Why do some top CEOs engage in serious leisure?” After a set of questions intended to assess the “seriousness” of their leisure interest, I continued with open questions such as “What does this activity do for you?” and “What does it bring you?” While I did not ask direct questions about stress management nor about the demands of the top job, I believe that this approach elicited more genuine insights into the CEOs’ struggles with their role demands (as the CEOs offered them unsolicited) than a more direct approach would have done [see Harding (2014), for an example of positive managerial talk obstructing the usefulness of research interviews]. The protocol was followed loosely, allowing and encouraging the CEOs to engage in storytelling and to freely associate starting from the topic of their passionate non-work interest.

The 16 interviews lasted between 27 and 93 min, with a median of 45 min. The fact that the interviewer is herself a former corporate CEO as well as a “serious leisurite” (a marathon runner) helped build trust, a condition for accessing “the inner world (meanings, ideas, feelings, intentions) or experienced social reality of the interviewee” (Alvesson, 2003: 16).

### Data Analysis

In analyzing the data, thematic analysis was used (Braun and Clarke, 2006), a method that, at its most basic, consists of the identification of patterns (“themes”) in a data set, followed by the analysis of their meaning and importance. More specifically, this paper uses “reflexive thematic analysis” (Braun and Clarke, 2019), thus named in order to distinguish it from more positivist forms of thematic analysis that are grounded on the assumption that there is a “truth” waiting to be discovered in the data and that the researchers should aim to stay as neutral as possible in order to extract that truth in their findings. By contrast, when engaging in reflexive thematic analysis, the researchers are keenly aware of their own role in developing the themes from the data and engage in constant reflection on the research process accompanying the recursive generation of the themes. To begin, reflexive thematic analysis requires that the researchers make (and state) several important choices (Braun et al., 2016). The first such choice regards the epistemological and ontological paradigm (e.g., realist, critical, constructionist, *etc*.). This paper’s epistemology is social constructionist, recognizing that leadership, executive work, executive stress, and the meaning of identity-shaping activities such as serious leisure are constructed and co-constructed by the main actors and their role partners in social interaction (Fairhurst, 2009). The second choice regards the use of a “bottom–up,” inductive approach whereby the data drives the coding and theme development and often even leads to a re-formulation of the research question *vs*. a more “top–down,” deductive approach whereby the analysis is, to some extent, informed by pre-existing theoretical concepts and driven by the research question. As this study’s research question was informed by the JD–R model combined with the concept of executive job demands and with the leisure recovery literature, and as in turn, the research question drove the analysis, this would indicate a mostly deductive approach. The third choice refers to awareness about how one engages with the data: at the “semantic” level, “coding explicitly stated ideas,” or at the “latent” level, reflecting “the meanings and frameworks that underpin the things explicitly stated” (Braun et al., 2016, p. 192). This paper aimed to engage with the data mostly at the latent level. However, as these choices are not either/or ones, most thematic analysis works include a mix of semantic and latent and, respectively, of inductive and deductive approaches (Braun et al., 2016), and the same is true for this paper.

The data analysis process followed the six steps outlined by Braun and Clarke (2006). Throughout this process, I was aware that as the interviewer always plays a constitutive part in the co-construction of “what is being said” together with the interviewee (Alvesson, 2003), my background as a CEO and an amateur marathon runner colored how the themes were ultimately developed.

The first step was deep familiarization with the data by listening to the interview recordings again and by reading and re-reading the interview transcripts. This step also refreshed the recollection of the face-to-face interaction with the CEOs as the tone of voice and/or the facial expression accompanying an otherwise apparently blank statement by the interviewee sometimes gave it a deeper meaning. For example, when the CEO whose pseudonym is Jason said, in his sparsely decorated office, “there is something about cycling that reminded me of my youth. Of that freedom of just…” and stopped abruptly, his voice and body language suggested the phrase could have ended with something like “riding away from all of this,” a latent-level message about yearning for freedom from the constraints of the CEO role. In this stage, I also started taking notes about what ideas may be in the data and why they might be relevant to the research question.

The second step was generating initial codes, “the most basic segment, or element, of the raw data or information that can be assessed in a meaningful way regarding the phenomenon” (Boyatzis, 1998, p. 63). Thus, the entire data set was systematically evaluated, and each data element was given full and equal attention in an iterative process of identifying aspects that may group into patterns across the whole data set that would later become the themes.

The third step was searching for themes. In this phase, codes were analyzed and tentatively grouped into combinations that shared a central idea, broadly guided by the research question. There were several iterations between steps two and three as thinking in terms of themes led to renaming, consolidating, and/or discarding some of the initial codes. Several dominant themes (and the codes underlying them) were identified, and then a number of “sub-themes” and their corresponding codes were also developed. To select the dominant themes, I used their relevance for the research question as the decisive criterion rather than how often they occurred or how much space they occupied in the total transcript. At this stage, I drew an initial “thematic map” (Braun and Clarke, 2006, p. 90) illustrating the themes, sub-themes, and the connections made by the interviewees between them. Finally, the remaining codes that did not fit into any of the themes were organized into a “placeholder” group. As I later iterated between this step and the next, some of the “placeholder” codes were later re-grouped into viable themes, while others were eventually discarded. The final list of underlying codes counted 59 such items capturing separate and relevant pieces of information.

**FIGURE 1:**
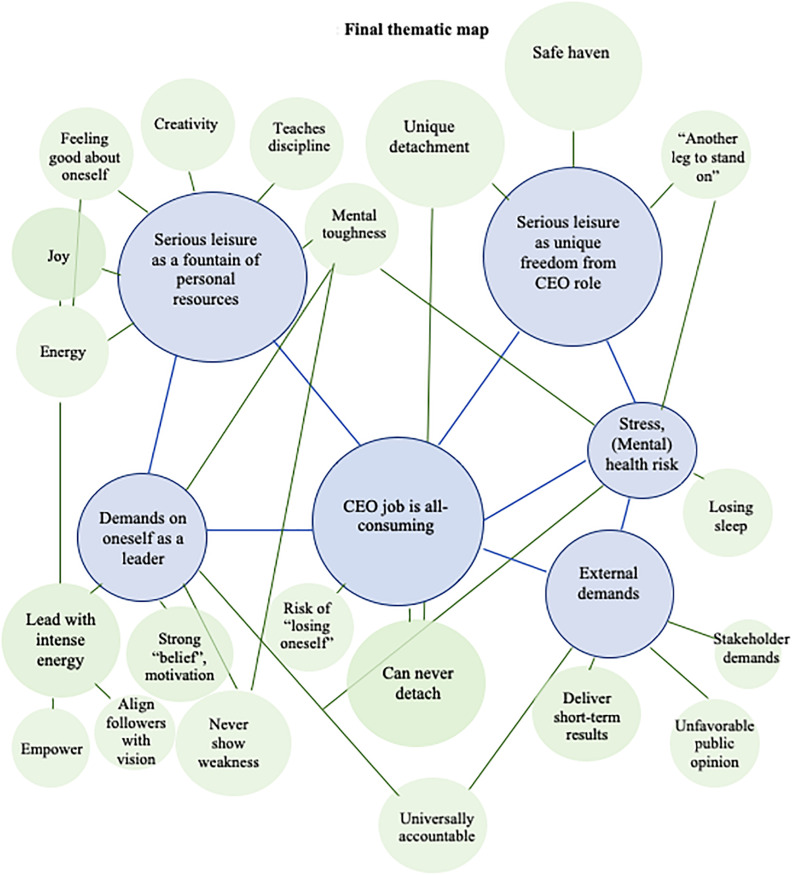
Final thematic map.

The fourth and fifth steps were represented by reviewing the themes, followed by the naming and the refining of the themes. Theme reviewing was undertaken with two aims: first, to assess whether the codes grouped under each theme really represent a coherent pattern and, second, by taking more of a “helicopter” view and re-reading the whole data set to gauge whether the themes generated really represented the data set completely and faithfully. The final six themes and 21 sub-themes were named and refined to represent the core of what each theme said about the data. The final thematic map is shown in Figure 1.

The sixth and final step was represented by the writing of the findings organized around the key themes and their sub-themes. The section below details how these relationships unfold.

## Results

Six key themes were developed around how the interviewed CEOs speak about the role of their serious leisure in responding to the demands of their job. The sequence in which they are presented is mostly determined by readability as, in reality, some of the CEOs started by speaking about the benefits of their serious leisure and then connecting them to the demands of their job, while others began by describing the all-encompassing nature of the CEO job before going into how their serious leisure helped them cope with it. These findings show that serious leisurite CEOs list coping with the overwhelming demands of their job among the main benefits of their non-work interest and that they see a unique, irreplaceable role for their serious leisure in this respect. Beyond managing stress, they find an equally important role for their serious leisure in creating valuable personal resources that support the CEOs in meeting their own demanding standards of leadership.

### The “All-Consuming” Nature of the CEO Job: Demanding Constant Sacrifice

The CEOs saw the demands of the top job as specific, different from those of other jobs and taking a larger-than-life presence: *You know what the job of CEO of a large company is like. It’s quite consuming*, said Anthony, singling out the CEO job (and specifically CEO of a large company) as qualitatively different in its demands. They rarely described these demands in concrete terms such as taking 100% of one’s time or continuously claiming one’s undivided attention and preferred instead to use metaphors (“all-consuming,” “all-encompassing,” and “sort of impossible”) that suggested immense effort and sacrifice. Nonetheless, they saw this self-sacrifice as fully justified, part and parcel of what it meant to do one’s job well: *[The CEO job] is an all-consuming thing. And I think that, if you don’t see it as an all-consuming thing, it’s difficult to get the job done* [Richard]. Similarly, while they saw the impossibility of ever detaching from work thoughts as a heavy demand, they spoke of it with the undertone of a needed sacrifice, a confirmation of their fulfilling their duty in an exemplary manner. *I rarely ever shut off my mind to what’s going on in the business. [*…*] you’re always thinking about what’s the next horizon, what are we doing, do we have the right people in the team* [Larry]. In the same manner suggestive of self-sacrifice, they described the loss of quality family time as their mind stayed in the office: *For me, coming home [*…*] there’s really not, as much as anybody would tell you there’s sort of an ‘off’ button, there really is not an ‘off’ button. It just goes on* [Anthony]. They admitted to trying to create the appearance of being fully present at family dinners or during holidays with loved ones while actually mulling over issues at work: *Even though it was meant to be a vacation [*…*] I’d go and hide in the toilet because she [interviewee’s wife] wouldn’t allow me to do this, and I would check emails, right?* [Richard].

The intensity of this “all-consuming” nature of the CEO job was further accentuated when the interviewees felt the price they paid sometimes went beyond giving up all of their mind space, all of their family time, to sacrificing their very self, and “losing” who they really are: *You realize you are just not yourself. And I see that happening to all executives at some point in their life. Because it’s a hard job* [Bill]. What they suggested was that their CEO role left little, if any, space for their personal, true self, an undertone so much more earnest when coming from people at the top of the corporate layers, conditioned to speak and think in positive, managerialist terms: *When you do this type of job, you are giving up so many things about you, your life, who you are* [Laura].

When attempting to further clarify what made their top role all-consuming, our interviewees spoke of demands broadly falling under two main themes: external demands and their own demands from themselves as leaders.

### External Demands: Delivering “The Impossible”

This paper’s interviews were held during increasingly difficult times for major company CEOs. On the one hand, public opinion was seeing corporations, as embodied by their CEOs, as driven exclusively by profits at the expense of society and the environment (e.g., Girouard, 2018). On the other hand, shareholders were growing impatient with regard to delivery of short-term results and, as they held the CEOs largely responsible for their companies’ performance, they tended to decide to replace them quicker than before, with record CEO turnover levels registered among S&P 500 companies (PWC, 2019). The fact that 2018 was the first year in which disclosing the “CEO pay ratio” became mandatory for public companies in the United States, leading to public outcry and, for some CEOs, to grueling sessions in Congress on the subject, did not help. Although these topics were not explicitly discussed with the CEOs in our sample, they were likely to represent a salient background in their minds when the topic of external demands placed on the CEO arose. Indeed the CEOs did broadly allude to the unfriendly external environment: *It’s a very tricky time right now for public companies and I think shareholder activism looks for*…*you know, under the guise of value creation, they look for chinks in the armor. They look for weaknesses* [Anthony]. Our interviewees felt that the multiplicity of stakeholder voices had dramatically increased over the past decade, often clamoring for contradictory demands: *There are too many constituencies, this is a big public company so there’s Wall Street, there’s the board, the shareholders, the employees, the customers, [*…*] there’s the community part of the job, I mean it’s sort of impossible* [Richard].

The interviewees underlined that the pressure to deliver short-term results had been increasing, at the expense of long-term strategic thinking: *They [analysts] are so much into the short term results that you can’t get them to think about what you’re trying to do over longer time horizons* [Jack]. At the same time, they expressed a realistic stance about how much it was in their power to achieve success, contrary to inflated stakeholder perceptions on this topic: *Luck plays a big role in a company’s performance. There is a perception of the CEO making or breaking it, when it’s really a lot about luck* [Thomas]. This humble view of their own abilities, echoed by many of our interviewees (*I cannot predict, I mean I fail on predicting like our business level because I just, I’ve been wrong so many times* [Jason]), comes in stark contrast with perceptions of today’s CEOs as markedly more narcissistic not only than the general population but also than the corporate chieftains of several decades ago (Hambrick and Wowak, 2012).

Despite the limits they saw to their abilities and power, the CEOs knew that they were held universally accountable for anything and everything happening in their vast, tens of thousands-strong and globe-wide spread corporations. This burden was not lightened by the fact that it aligned with their own beliefs about a CEO’s responsibility: *You’re accountable for everything that happens in your business, your track record, performance [*…*] And if we have an environmental issue in China, I’m ultimately accountable, and I should be, that’s what the role of the leader is* [Larry]. The theme of the interviewees’ own expectations from themselves as leaders represented another heavy demand.

### Demands on Oneself as a Leader: “Grace Under Pressure”

What arose as a source of internal pressure for our interviewees was living up to their own high standards for how a leader should behave, around sub-themes such as inspiring, creating, and maintaining alignment and belief in a common vision, empowering one’s teams, never showing weakness, and especially having, showing, and using up immense stores of energy.

Inspiring one’s followers was seen as a key condition for leading well as a CEO, qualitatively differently from leadership in other roles: *I’ve really learned, since becoming a CEO in another company for a couple of years and then coming here, that a big part of the top job is not just to communicate with people, but actually to inspire them, to make them believe in what we are putting together* [Thomas]. How the CEOs spoke about inspiring and creating alignment conjured up the image of a continuous effort by the leader, never completed, burning massive amounts of personal energy: *Even people in a company with a purpose as clear as ours, which you’d think they’d wake up every day ready to go get it, but they feel all the weight and pressure and distractions and frustrations, and they do have to be reminded periodically, through my own energy and passion, that there’s a larger purpose here* [James].

Energy was seen as crucial by many of our CEOs, not only as a personal resource that gets heavily tapped into by the demands of the job but also as the very blood flowing through the organization’s veins, a *sine qua non for* the organization’s success: *We’re making commitments, I blew mine up and put it on my office door so people can see what I’m focusing on. Number one is, that I commit that I’m going to manage the organization’s energy. Are we inspiring [*…*]?* [Thomas]. Similarly, the interviewees believed that they allocated large amounts of energy to achieving the difficult task of truly empowering their teams. Empowering, giving one’s employees the space to take their own initiatives and make their own mistakes, is seen by academia and practice alike as one of the basic elements of good leadership, something one would expect to master early on in one’s leadership journey (e.g., Kouzes and Posner, 2017). This made the CEOs’ confessions about how difficult they actually found this in practice all the more powerful: *I need to do a better job of letting them do it. And letting them experience without my involvement. [*…*] And it’s hard, I mean there’s times when I just want to jump in and grab the wheel. Because I can see, I can see that what they are doing is not going to work* [Bill].

The CEOs expected of themselves to constantly control their emotions so as not to show weakness such as doubt, worry, or discouragement (or, as Susan put it in mantra-like form, *show grace under pressure* and *never let them see you sweat*). They saw the role of the leader as projecting unabated calm and confidence in the most difficult of crises: *I never told anybody I was scared, but I was scared. Because I wasn’t really sure what to do. [*…*] Your team doesn’t want to hear you say you’re scared. They want to hear you are not scared. That everything is going to be OK* [Bill]. Although a discourse on the power of owning up to vulnerability has increasingly become popular in the corporate world and several of our CEOs actually referred to the value of showing vulnerability, they conscripted this notion to a well-defined range of emotions and self-disclosure that would not include, for example, showing doubt, exhaustion, or fear despite their experiencing these emotions in their leadership role. The CEOs also demanded of themselves to maintain unwavering belief in the long-term vision, even in the absence of tangible progress: *It always takes longer than what one thinks it’s going to take, and as it takes time you begin to doubt yourself […] as in ‘are we not getting there as fast as we should’ because maybe I’m not right? Maybe I’m missing something here* [James]. In sum, they fell prey to “romance of leadership” notions (Bligh et al., 2011) by expecting of themselves to be supremely and unwaveringly inspiring, energetic, and optimistic leaders, although they had earlier criticized similarly romantic expectations of external stakeholders regarding a CEO’s power to single-handedly determine the results of the organization.

We next connect the “all-consuming” nature of the CEO job and the related themes of external and self-inflicted pressure, with the key theme of stress and mental health risk.

### Impact of CEOs’ Job Demands on Mental Health: “A Good Chance of Cracking”

Most of our serious leisurite CEOs spoke about the impact that this “all-consuming” nature of the CEO job could have on their levels of stress only indirectly, when they illustrated how their serious leisure protected them against significant risks in this respect. However, a telling illustration of how serious stress levels can become in the absence of an outlet such as practicing serious leisure was offered by Laura, who, at the time of our interview, had just re-started practicing horse riding, a life-long passion, after several years of pausing it in order to dedicate herself completely to her (first-time) CEO role, as she realized that reconnecting with her serious leisure had become *a matter of survival* [Laura]: *I got to a point where I’m thinking about work 100% of the time. I’m actually almost not sleeping anymore*. Similarly, Richard described a particularly difficult period as: *Stress was so big that I would sleep very, very poorly*. Other CEOs preferred to speak about excess stress as a general risk of the job rather than their own experience, a chilling reminder of how serious the mental health risk of the top job could become in the absence of resources such as those brought by serious leisure: *The board members [*…*] they are aware that you need recovery, you need some balance, they don’t want to find you one day with a gun on the top floor* [Thomas]. *I think that if you don’t have something like this [a serious leisure interest] you have a very good chance of cracking!* [Paul].

Among the benefits our CEOs found in their serious leisure interest, two main themes emerged, which are connected to the subject of responding to the demands of the CEO job: first, serious leisure as freedom from the CEO role and, second, serious leisure as a fountain of personal resources.

### Serious Leisure as Freedom From the CEO Role

Our interviewees spoke about their passionate non-work interests as a precious source of freedom from the “all-consuming” CEO role. They consistently identified their serious leisure as the only activity able to bring them detachment from work thoughts: *When I was going through this difficult [work] situation, not that long ago, I was actually surprised, like, when I practiced tennis [*…*] that I was gone, I was gone, I was really*…*So it takes your mind off. And it’s much better than taking drugs or getting drunk* [Paul].

While the fact that leisure can offer detachment from work rumination is hardly surprising, what the CEOs underlined was that no other non-work activity succeeded in offering them respite from job-related thoughts, which made serious leisure unique: *In other exercise that is non-competitive, non-team based, oh, my mind immediately goes to work* [Steven].

At a deeper, more latent level, the CEOs seemed to need their leisure to be “serious” in order to balance the drive to sacrifice their whole time and mind space to work. A serious leisure interest, through the intensity of the activity and the “pull” the passion for it exerted upon its practitioners, would be the only one able to “force” the CEOs to dedicate time to it and to forget all about work while doing so: *It [woodwork] forces you to take your mind off, off that employee who had a problem, off that board discussion*…*it quiets your mind that way* [Thomas]. *What I like about flying is that it takes so much concentration that you’re really not able to think about anything else* [Bill].

The CEOs sometimes spoke of serious leisure as “me time,” a rare occasion for them to enjoy doing something for themselves, away from the duties of their role: *I do [greeting cards] over the weekend, I typically open a bottle of wine, I light a candle and, you know, I come into my den and I spend an hour or two making cards. It’s my release, my hobby, there’s no one here, no interruptions. And it’s very peaceful* [Robert], but when job demands became even more intense, nearing burnout, such as in Laura’s case, *Would I do the same thing (take the CEO job) again? I probably wouldn’t actually. [*…*] Because it’s very difficult*, or at an earlier time in Richard’s CEO life, their serious leisure became more than “me time,” a “safe haven”: *This [soccer] was like a safe haven [*…*] it’s like the one safe place where I think the stress, you know, it’s sort of helped control things* [Richard].

For Laura, her horse riding was a safe haven not only due to the rare peace of mind it offered but also because it represented a separate world where one could freely express oneself away from the constraints and the pressures of the CEO role: *This is my world. This is my world. The horses, the horse world is my world* [Laura]. Many of our CEOs subscribed to this view of their serious leisure as a separate place for self-expression, a place to re-affirm that “true self” they had felt was being crowded out by the all-encompassing CEO role: *The second I got back on stage, I felt like I was home again*, said Anthony who, after taking a long break from performing in public as a singer–songwriter, finally decided to restart performing (for charity) while CEO of a major listed company. In this separate place, our interviewees cherished not being seen as CEOs, specifically in order to reaffirm their value as individuals outside the CEO role: *The minute you walk in, everyone’s the same [*…*] And for a long time, most people didn’t know what I did, which was awesome!* [Mary].

Some of our interviewees went further in the self-reflection exercise occasioned by our talk and identified the crucial importance of a non-work passion as “another leg to stand on,” a way to diversify from the overpowering CEO identity so that they would not take setbacks in their work role as a disastrous reflection on their whole self-worth: *I’ve had massive disappointments in my business life, was massively disappointed about my results. […] And that’s where I believe, to have a second leg to stand on, to have another life outside of your profession, I think that’s so important, to get through those valleys. You screw up something in your business life [*…*] but then you go “well, I’m not a failure! I screwed up here yet as a person I’m not a failure, because I know I’m a pretty good painter! I’m a pretty good*…*whatever, right?* [Paul]. Being able to define themselves through more than the CEO persona, specifically through a self-enhancing, positive identity such as that provided by a serious leisure interest, thus offered another dimension of welcome freedom from the CEO role.

While much of what has been presented so far regarding serious leisure’s benefits has referred to mitigating the strain arising from the CEO’s job demands, the interviewees also saw the “freedom from the CEO role” offered by their serious leisure as a source of personal resources that promote optimal functioning in the demanding CEO role. *I’d rather spend more time away from work in order to do a better job at work*, said Jason, pointing to the quality of his decision making, as well as his creativity: *I think it’s just important to have something else. Because I can’t process just linearly doing work, I need some other stimulus* [Jason].

However, the CEOs also believed that their serious leisure directly created a wealth of personal resources that were needed to respond to their own expectations about performance in the leader role.

### Serious Leisure as a Fountain of Personal Resources

One of the strongest benefits of serious leisure that the CEOs identified was its ability to renew and increase their “energy.” I interpret their understanding of “energy” as broadly similar with the concept of psychological capital, which is also consistent with recent Psycap studies (Rego et al., 2019). However, to stay close to the interviewees’ voices, I will continue to use the term “energy” throughout this paper. The CEOs found a strong source of energy in practicing their serious leisure and, as mentioned earlier, they considered having and giving energy as a *sine qua non for* leaders: *If you, as a leader, aren’t able to find that right balance yourself, and always be able to create energy and focus your own energy, and conserve your energy, then at some point in time, it’s inevitably going to impact the organization you’re leading. So that’s the big role the music plays, it feeds me in terms of making me happy, or allow me to release tension, it’s just, it recreates positive energy* [James]. They often realized how important their serious leisure was to their energy levels especially when they were forced to slow it down: *A year and a half back there was a two-month period when my running actually dropped because we were in the middle of a couple of big acquisitions. [*…*] That actually, I realized, impacted me because I didn’t feel that energetic* [Kenneth].

This energy was associated with the intense joy our interviewees found in their serious leisure. Asked what ice hockey brought him, Steven illustrated a full trajectory of positive emotions, from anticipation to enjoyment to happiness to optimism: *I’d go with joy! Driving to it, I look very much forward to getting there and thoroughly enjoying myself throughout. [*…*] I am a happier person when I have finished the hockey game and showered up and got into my car, I am ready for whatever comes next* [Steven]. *I just love golf*, says Kevin playfully and he repeats it 27 (!) times during our interview.

Our CEOs’ energy boost also came from feeling proud of their achievements in their serious leisure pursuits. These men and women who command corporate empires do find a special sense of pride and mastery in their personal pursuits, and this is not limited to the competitive, athletic pursuits either: *So when I inventory why I do it and what I get from it, I really get tremendous satisfaction in the creation of a one-of-a-kind message [on greeting cards]. I think, in some ways, people think my business success is the thing I’ll be most remembered for. I don’t think so, although we were very successful. I think it will be the sticker cards* [Robert]. About his woodwork hobby, Thomas beamed: *It does give me a sense of pride, I have sometimes even sold some of the things I made, auctioned them for charity* [Thomas]. As earlier mentioned, this self-affirming serious leisure identity can play a unique role in offering “diversification” from the overpowering CEO identity.

Our interviewees saw their serious leisure as fostering creativity [*It gives me that time to let my mind somewhat wander [*…*] and it’s amazing because problems that I didn’t think that I could solve, some sort of new insight will come to me* (Susan)] and sharpening their “mental agility”: *If in a particular month instead of 200 miles I run 75 miles*, then *that month is not a good month. I don’t feel good. [*…*] If there are sustained periods when I’m unable to actually do that level of physical activity, I find my mental activity and mental agility do down*, says Kenneth, thus distinguishing between mere exercise and the superior powers of his serious leisure in offering the mental agility required by the demands of the CEO job.

Kenneth’s belief in the special value of a 200-miles-per-month regime exemplifies the multitude of narrative, metaphorical sensemaking that our interviewees engaged in, regarding the abilities of their serious leisure to support them in meeting the demands of their role. From an “objective” standpoint, the value of more moderate exercise (say, 100 miles per month) for mental agility and energy level could be judged as higher than more “extreme” exercise alternatives. However, what is important here is the story Kenneth tells himself regarding the unique value of those 200 miles per month. Another such narrative several of the CEOs told themselves was that their serious leisure had taught them that consistency of effort leads to results. They found that they successfully applied this lesson in their leader role where, as we have seen, they saw belief in the long-term plan and vision as a crucial part of leading as a CEO. Thus, using what their serious leisure had taught them, the CEOs moved their focus from the uncertain results of their long-term strategy (says Laura: *when my people are talking about numbers, money, turnover, I say ‘Don’t bother about this. This is the outcome of good work’*) to the more controllable input: *There’s this discipline where you keep doing it with the belief that eventually it will pay off. For the Death Ride [*…*] my goal was to finish in under 9 h of ride time. So I mapped that, the training of that, for months. [*…*] And the ride was then, on a 9-h ride, I was within 3 min of what I had planned. [*…*] With work it’s the same way. A lot of the projects that you do have to be over a long period of time, so this consistency of effort, the metrics that you have, I think the mindset is very similar*” [Jason], or as Larry put it: *[football] teaches you, if you train and develop every day in the grind, and if you do the right things, success will eventually come* [Larry].

Thus, the CEOs found that the regular effort they put in their serious leisure helped them get a sense of control in the midst of uncertainty. As Jason continued: *I got into cycling right after the financial downturn. And a lot of it was: ‘I can control this! I cannot control the world, but I can control how I exercise. And I need some level of control over something’. And this job, there’s so much that you*…*I don’t know what kind of trade war we are going to start. I’ve no idea, but I do know that, that part of it, having some control over something* [Jason].

Similarly, Bill believed that during a “scary,” crisis time in a former CEO position, his sports mindset came to the rescue by reminding him to focus on what he could control, his own effort: *I was taught never to give up. I was taught that you work as hard as you can, as fast as you can, until the coach takes you off the field. So I woke up every morning and I thought to myself ‘the coach hasn’t taken me out yet. So I am going to go and do the very best I can. And if what I’m doing is not good enough […] then the coach should make a change. If I’m not effective as [name of company]’s leader […] then obviously they should change it. […] But until they tell me that, I’m going to do the best I can* [Bill].

The CEOs also believed that their serious leisure had educated in them and helped them maintain the “mental toughness” that is required to withstand the pressure of the top job, especially when self-doubt beckons: *Athletics teaches you how to persevere [*…*] you have to be able to deal with the setbacks and pitfalls and*…*life is not perfect, how to recalibrate, rethink what you’re doing. [*…*] Because if you can’t overcome mentally the challenges, you are not going to be successful. I don’t talk about mental toughness in business, maybe I should […] how do you build your mind to overcome that self-doubt; and everybody has self-doubt* [Larry]. As Mary said: *Confidence to me breeds resilience, and resilience then means that you can withstand a level of pressure that other people may not [*…*] [Martial arts] is definitely a confidence builder* [Mary].

In conclusion, our interviewees believed that their serious leisure helped them cope with the strain caused by the intense demands of the CEO job by offering them freedom from the CEO role and by creating a wealth of personal resources that would, in turn, support them in rising to their own standards of leadership in the job. Table 2 presents additional support for these findings.

**TABLE 2:** Themes, sub-themes, and illustrative quotes.

Themes and sub-themes	Illustrative quotes
**CEO role is “all-consuming”**	
Can never detach	“This job can be very time-consuming and all-consuming […] You talk to a lot of CEOs, you’ve been one, it’s all-encompassing” [Larry].
	“You’re always, always, always thinking about work” [Richard].
Risk of “losing oneself”	“The whole thing is, I’m actually a very private person and […] I did not want myself to be the center of attention […] and reality is, there’s a price you pay for that, right?” [Richard].
	“Sometimes I think I should have stayed in coaching and teaching” [Larry].
**External demands**	
Universally accountable	“The board does have an expectation that is fully justified, that you are fully committed to the job and that you’ll be there for it” [Thomas].
	“The amount of responsibility you have is fundamentally different than other people’s” [Mary].
Deliver short-term results	“In the US they tend to have shorter time frames. And expect instantaneous results. And if you’re not successful within a short period of time you get fired” [Richard].
	“It’s funny, we talk about shareholders, I think that it’s a mythical shareholder. Because the actual shareholders don’t tend to hold stock for very long […] But in the company you really try to optimize for the longer-term shareholder, which is a theoretical idea” [Jason].
Stakeholder demands	“The external stakeholders, the analysts, are pretty critical to the CEO, they’re really your customers and you’ve got to treat them like customers. […] Because they can tear you down a lot faster if they choose to” [Larry].
**Demands on oneself as a leader**	
Strong “belief,” motivation	“You need to develop an inner confidence that ‘hey, it may not feel good right now, it may not feel like it’s going the way I wanted,’ just give it some time!” [James].
	“From the time that you make the investment until you see the positive financial result it’s sort of a minimum of a 5-year time frame, and you have to sort of be very strong in your conviction” [Richard].
	“When you become a CEO you have to rely completely on yourself, to motivate yourself, to motivate your company, your clients and everyone around you. And that’s totally on you” [Mary].
Never show weakness	“If you want to control chaos […] find that purposeful power within yourself, that gives you that true ‘sense of grace”’/“Under that stress, under those situations, to remain calm, to just kind of set your stride and keep moving” [Susan]. “Everybody has self-doubt. The big part for me is, when you feel like that, and we all do, periodically, you can’t portray that in the organization, you have to keep that same strong approach” [Larry].
Align followers with vision	“Alignment is the single most underrated success factor of leadership and you don’t get, you can’t fake alignment, you’ve got to earn it, and I think many CEOs and leaders don’t fully appreciate that” [James].
Lead with intense energy	“I’m a big believer in energy management. […] energy has a big correlation with your results and impact” [Paul].
	“One of the most important things that any leader does […] is how they create energy for the organization and how they focus and manage the energy of the organization. And if you, as a leader, aren’t able to find that right balance yourself, and always be able to create energy and focus your own energy and conserve your energy, then at some point in time it’s inevitably going to impact the organization you’re leading” [James].
Empower	“One of the changes I had to make, which was difficult but necessary, was […] change the leadership to go to the next generation of the leaders underneath me […] So then, how do you change in terms of areas that you can provide guidance with, and yet how do you not let go so much that they make mistakes, or what I perceive as mistakes” [Jason].
**Stress, (mental) health risk**	
	“The CEO job is very stressful” [Richard].
	“I’m not sure it’s helping to be a public company CEO for too long because it’s not the most…it’s a stressful environment” [Bill].
	“Stress management, right, in these jobs, if you aren’t fit, it takes a toll, and it takes a hard toll” [Jason].
	“I will ride three times this week, there are only those 3 hours when I’m absolutely not thinking about work. Which I think is completely required. It’s almost a survival decision for me right now” [Laura].
**Serious leisure as freedom from the CEO role**	
Unique detachment	“Even if I’m stressed out about anything that’s going on at work, and I have a lot on my mind going into [martial arts] class, by the end of class my mind has just kind of released itself, because once you start class, you really don’t think about anything else! So it’s a very good way to release the stress” [Mary].
	“I started to ride more intensely again […] a month ago, yeah, because I thought I need to do something that would really help me to… It’s almost like meditation, you know, when you ride. […] When you ride you can’t think about something else. It’s impossible” [Laura].
“Safe haven,” a different world	“I start out selfish every single day […] before life takes over in the day you start and do something for yourself” [Paul]. “Music is such a big part of who I am, that if it meant playing at one in the morning, when everyone else is sleeping, and sitting at the piano for 45 min, I was totally fine with that” [Anthony].
	“It is a very close-knit community because most people don’t understand what goes into coaching in time, commitment” [Larry].
It is “another leg to stand on”	“When I fly, a very different person comes out” [Kevin].
	“Because I have these other aptitudes, I can feel like I failed at something or I was disappointed in myself at something without feeling like a failure […] because our stock dropped on a certain day, I can feel disappointed in how I handled it, but I don’t doubt my value as a human, because I know that I’m a composite sketch of a lot of things of which this is a very important part, but not the whole me” [Anthony].
**Serious leisure as a fountain of personal resources**	
Energy	“When I have finished the hockey game and showered up and got into my car, I am ready for whatever comes next” [Steven].
	“It gives me a lot of energy” [Mary].
Joy	“The actual creation of a card is maybe my greatest personal pleasures” [Robert].
	“It gives me a great, great deal of happiness. So I’m really happy when I’m flying an airplane and I’m also happy with the anticipation of flying an airplane. So it gives me something to look forward to and it’s a real joy, it’s a real pleasure” [Bill].
Makes me feel good about myself	“It [flying] also gives me a sense of pride and accomplishment, I’m proud of the fact that I’m able to do that. It makes me feel good about myself” [Bill].
	“Not many people have a pilot’s license and I just like the idea of being able to do something that most people on the planet can’t do” [Kevin].
Builds creativity, mental agility	“I am an engineer by education and I like making things and creating things. And in this job it’s obviously more about inspiring others but I miss the creativity part. And the woodwork, that’s what it brings back, and sometimes after doing it I find my creative juices flow more freely” [Thomas].
Discipline, sense of control	“I’ve always felt I was a very good planner, that I was very good at anticipating things, and I really think my experience with flying has really, either developed that, or made it stronger” [Bill].
	“It gives you that freedom, that sense of purposeful power […] that true sense of control” [Susan].
Tenacity, “mental toughness”	“I’m running, biking, I’m working really hard and it’s kind of pushing to the limit and pushing kind of beyond what I think I can achieve, and that will go over to work” [Susan].
	“I’ve always thought that golf was, in some ways, symbolic of life. […] I think that, to get through a round of golf, it’s mainly mental. […] I think the biggest difference is the mental toughness and the ability to navigate all the things that happen to all of us, in real life” [Bill].
	“When I think of music, it’s got some similarities [with my work]. There is no shortcut. Like, you can have musical aptitude, but you need to put in the hours. You need to train” [Anthony].

## Discussion

This paper provides a rare glimpse inside top CEOs’ sensemaking with regard to the demands of their job as well as a first exploration of how a specific use of their limited spare time, engaging in serious leisure, helps them cope with those demands. Our findings are noteworthy not only because CEOs’ mental health and optimal functioning are important to their own organizations but also because major company CEOs often represent role models for (aspiring) leaders throughout the corporate world. Moreover, for many of our interviewees, their practice of their serious leisure interest started long before they became CEOs, and their beliefs regarding the power of their serious leisure to create valuable resources are also grounded in memories from these earlier times and less senior leadership roles, which makes our findings relevant also for the broader study of leaders’ mental health and optimal functioning.

### Theoretical Implications

This study’s findings bring up several important novel insights that contribute to research on the buffering role of detachment from work and personal resources on the impact high job demands have on stress to the upper echelon (and specifically executive job demands) literature, to research on leaders’ personal resources, and to studies of work recovery through leisure.

First, while research has documented that psychological detachment from work (Sonnentag et al., 2010) helps buffer the strain brought by high job demands, the present study contributes to this literature a rare view of how, when it comes to CEOs, they perceive their serious leisure as one of the few non-work activities (if not the only one) that can achieve this beneficial effect of detachment, given the “chronic” activation of their CEO identity. Moreover, recent views are that “constellations of recovery experiences” will likely yield more than the sum of their parts (Bennett et al., 2016, p. 1635), yet there is little empirical research focusing on combinations of recovery experiences rather than on the effects of distinct recovery experiences (such as only psychological detachment or only mastery). This study shows how serious leisure can provide a potent such combination as all five main recovery experiences identified by prior research (psychological detachment, relaxation, mastery, control, and enjoyment) (Sonnentag and Fritz, 2007) are present in our interviewees’ accounts: thus, the “freedom from the CEO role” category contains important psychological detachment elements as well as relaxation elements (related to the “me time” and “safe haven” themes), while the “personal resources” category includes enjoyment, “feeling good about oneself,” an indication of the experiences of mastery provided by serious leisure and the “sense of control” that comes with putting in consistent and measurable effort for measurable results. This paper also finds that serious leisure creates powerful personal resources not only indirectly, through the recovery experiences it offers, but also directly, through the stories our CEOs tell to themselves about their serious leisure’s power to develop a mindset of “mental toughness,” of discipline, and of belief that consistency of effort leads to results. As leisure research indicates, beliefs about the stress-coping and flourishing benefits of one’s leisure are essential for these benefits to actually occur and act independently from what the actual engagement in leisure provides (Iwasaki, 2003). However, as these studies did not focus on serious leisure, these beliefs were limited to what casual/general leisure is thought to offer. As serious leisure is a passionate pursuit that its practitioners internalize as a strong personal identity and therefore a rich source of meaning (Allen-Collinson and Hockey, 2007; Falcous, 2017), they are bound to construct stronger and broader beliefs around it compared to casual leisure. Moreover, since the CEO job represents another salient role for our interviewees, according to the role enrichment theory, they will be motivated to transfer resources generated in serious leisure to the CEO role (Greenhaus and Powell, 2006). Our study brings a rare insight into how the strong serious leisure identity promotes CEOs’ meaning making around their serious leisure as a provider of abundant resources and its role in their optimal functioning in the CEO role.

Second, our study identifies how serious leisure can represent an antecedent of leader psychological capital (Luthans and Youssef-Morgan, 2017) that itself has been shown to correlate with follower performance (Chen, 2015) and follower work engagement (Xu et al., 2017). As recent research shows, conveyed leader psychological capital (also broadly referred to as “energy”) is especially powerful in generating positive follower outcomes and, at the same time, is most effective when aligned with the leader’s own perceptions of their psychological capital (Rego et al., 2019). This paper’s insights show not only how CEOs see the importance of conveying high personal energy to their employees but also how they believe that their passionate interests play a key role in helping them recover and increase their energy level.

Third, what the interviewees perceived as “freedom from the CEO role” offered by their serious leisure referred not only to its unique powers of detachment but also to its ability to offer “diversification” from an over-investment in the CEO identity or, in the words of one of our interviewees, “another leg to stand on.” A positive identity, not related to the other identities the individual has, increases the individual’s positive self-complexity, buffering their well-being when they are confronted with negative events in one of their other roles (Linville, 1987). Individuals high in self-complexity have a greater tolerance for frustration (Gramzow et al., 2000), have lower affective reactivity, and cope better with negative events (Koch and Shepperd, 2004) as setbacks in one of their valued roles (such as the CEO role) do not spill over to their whole self. Given the importance our interviewees attached to the CEO’s always being calm, optimistic, and in control, serious leisure thus proves to be a unique asset through the positive, agentic identity it creates and that casual leisure pursuits cannot offer.

Fourth, this paper responds to the call formulated by Hambrick et al. (2005a) who, after introducing the concept of executive job demands, saw it as a promising arena not only for organizational theorists but also for organizational behavior and applied psychology researchers focusing on the individual level. However, little, if any, empirical studies have since attempted to look inside the “black box” of executive job demands as perceived by the executives themselves. This study responds to this call by contributing a thick description of how the executives at the top of the pyramid, CEOs of large companies, perceive the demands of their job as arising both from external factors and from their own standards of leadership in the top job and how they reconcile the need for freedom from the CEO role with the duty that they feel to sacrifice their time, energy, and “true selves” to it by constructing their serious leisure as, on one hand, “forcing” them to break free from the CEO role and, on the other hand, supporting their top job by creating important personal resources. The CEOs in our sample perceived that they were sacrificing their whole personal, waking, and sleeping life for the larger-than-life CEO role, while at the same time they intuited the risks to their mental and physical health of doing so. Illustrating the well-worn adage “work hard, play hard,” they thus seemed to see their “play” interest as an equally intense and passionate counterbalance to the overpowering nature of the CEO role.

### Practical Implications

This study also holds several practical implications for top executives and their boards. First, although CEOs appreciate that, like any human, they need some downtime to recover from work, they allow very little time for leisure in their agendas (Lees, 2018). Thus, the question becomes, what type of leisure activity would be (a) a strong enough motivator to pry their hands away from the corporate steering wheel (that they otherwise cannot let go of or, at least, cannot stop worrying about, even when with friends or family) for the time needed for adequate recovery and (b) the most potent in terms of resource restoration and creation. A serious leisure interest that, as Jason put it, “forces a discipline to not always be working” seems to be one of the answers to this question. Thus, in the quest for optimizing their personal resources, CEOs would do well to consider reconnecting with a long-lost non-work interest they had once held dear or looking for a new one while staying aware that the motivation for developing it into serious leisure should come from within, from the joy and passion it ignites, not from instrumental reasons. For large company boards, paying attention to the CEO’s mental health is critical. While many of our interviewees mentioned that their boards had recommended that they work less or take more time off, there is little actionable content in these recommendations. Even when more specific, such as “get a hobby,” a familiar entreaty to over-committed executives, such a recommendation has had little research support so far. “Take a vacation,” another staple of well-intended advice to workaholics, may not always work, as exemplified by our CEOs’ confessions as they continue to ruminate about work (if not engage in work outright through online channels), only now in a changed scenery. Moreover, the recovery effect of vacations has been shown to be short-lived, with stress and burnout quickly returning to pre-vacation levels once employees are back at work (Sonnentag et al., 2017). It would also be desirable that, in the spirit of risk management that boards are acutely aware of, such recommendations should prevent rather than treat: while serious leisure is seen as a strong builder of resources against burnout, starting out in a serious leisure pursuit may be stressful in itself and take considerable time, possibly with some false starts as one identifies one’s true passion and typically with a steep learning curve for the pursuit’s specific skill, not something one would want to undertake when in or nearing work-related burnout. Thus, we hope to have offered company boards useful insights into how passionate hobbies can help over and above other recovery activities so that such future nudging of their CEOs can be timelier and more specific.

### Limitations and Future Research

As with all qualitative research, our study makes no claims to statistical generalizability. However, it is notable that the interviewed CEOs had relatively homogeneous views on the topics discussed, with the key themes being representative of all interviews. Where differences appeared, they were more of degree than of nature: for example, Laura identified higher levels of strain and of job demands than the other CEOs, but not qualitatively different components of either job demands or strain. This convergence of views would probably not surprise the CEOs themselves as several of them referred to the job of a large company CEO as posing specific challenges, thus suggesting at the same time similarity inside the “in-group” and distinctiveness from other types and levels of executive jobs. Moreover, it is possible that the fact that the interviewees are all “serious leisurites” may mean that they are more similar along certain traits (such as, for instance, conscientiousness) which may impact how they perceive strain and job demands (Hambrick et al., 2005a). All of the above suggest that outright generalization of this paper’s findings outside the population of large company CEOs who have a serious leisure interest would not be advisable. Nevertheless, the phenomenon of serious leisurite top CEOs is significant and growing, with over 10% of the CEOs of S&P 500 companies publicly known to have a serious leisure interest (Bunea et al., 2018), which, when adding the “silent” serious leisurite CEOs whose passionate non-work interest may have not (yet) attracted the media’s attention, probably puts the total percentage significantly higher. These individuals lead some of the largest companies worldwide and there are arguably thousands of individuals worldwide whose aspirations involve becoming CEO of an S&P 500 company. A thick description of these CEOs’ sensemaking around their serious leisure and their job demands is therefore valuable in itself in terms of its contribution to management research. However, this paper’s contribution may be larger as the thick qualitative insights it brings into these CEOs’ leadership and job demand challenges and constructions may foster representational generalization (Lewis, 2014), a type of non-statistical generalization that can be reached when the qualitative findings resonate with the reader’s own, direct or vicarious, experiences or, in our case, when an executive who is not a large company CEO would recognize their own engagement in life’s affairs in (some of) this study’s findings.

Our study does not dwell on gender issues, given the low representation of female CEOs in our sample (as indeed among all S&P 500 companies). However, serious leisure research shows that women have difficulty in accessing serious leisure due to the gendered expectations of their other role partners (Raisborough, 2006), as well as to the often masculine, exclusionist culture surrounding many leisure pursuits (Anderson and Taylor, 2010). Future research could focus on women leaders and their serious leisure and on the specific strategies they use or have used to gain, from others and from themselves, the right to have an ostensibly “selfish” avocational interest.

In our study, we aimed to use a broad lens, without differentiating between types of serious leisure (such as sports *vs*. music or arts and crafts) and focusing instead on the common themes brought up in our interviews. Leisure research has shown that attempting taxonomies based on “objective” characteristics of various types of leisure is not desirable as what unites various leisure interests is how they are experienced by the participant rather than externally observable characteristics (Banner, 1985). Indeed our study shows that the fact that these apparently very distinct interests are experienced by our interviewees similarly, leading to strong identification and feelings of mastery, self-expression, self-actualization, and joy, means that what they share is stronger than what differentiates them. Still it is undeniable that, for example, sports have specific physical benefits for work recovery, and therefore we can benefit from dedicated future explorations of how sports practiced as serious leisure benefit leaders’ coping with their job demands. One should tread carefully though as what differentiates serious leisure is precisely the fact that it is freely chosen and intrinsically motivated, and therefore any attempt to recommend specific types of activities, such as sports, to be practiced as serious leisure by leaders could lead to increasing, instead of decreasing, the overall strain experienced by the leader.

## Conclusion

This study has explored serious leisure participants’ perceptions with regard to how their non-work passion supports them in responding to the demands of their job at a rarely accessed level: CEOs of large companies. Its findings offer new insights into how serious leisurite CEOs perceive the demands of their job as arising both from external and from self-inflicted pressure and how they believe that their serious leisure plays two distinct and uniquely valuable roles in helping them respond to these demands: one in offering them freedom from the overpowering CEO role and the other one in helping them build strong personal resources needed to rise up to their own expectations of leadership in the top role. Responding to calls for more empirical investigations of CEOs’ emotions and cognitions, we hope to spark further interest in the specific, growing phenomenon of top leaders’ serious leisure, to the benefit of tomorrow’s leaders and organizations.

## Data Availability Statement

The datasets generated for this study are not openly available due to strict participant privacy conditions. Requests to access these datasets should be directed to the author ate.m.bunea@vu.nl.

## Ethics Statement

Ethical review and approval was not required for the study on human participants in accordance with the local legislation and institutional requirements. The patients/participants provided their written informed consent to participate in this study.

## Author Contributions

The author confirms being the sole contributor of this work and has approved it for publication.

## Conflict of Interest

The author declares that the research was conducted in the absence of any commercial or financial relationships that could be construed as a potential conflict of interest.

## Footnotes

1 Of the thresholds available on Linkedin for filtering based on headcount, a minimum of 5,000 seemed most likely to yield companies comparable in headcount to those in the S&P 500 index rather than the neighboring options of 1,000 and 10,000.

2 According to the companies’ websites, more accurate than the headcount information provided on Linkedin.

3 Source: Compustat, author’s calculations.

4 Financial services, technology, electronics, science and technology, restaurants, business process outsourcing, retail stores, professional services, industrial goods, medical services, industrial packaging, health insurance, health services, and home improvement.
